# Comparison of the Effects of Maternal Supportive Care and Acupressure (BL32 Acupoint) on Pregnant Women's Pain Intensity and Delivery Outcome

**DOI:** 10.1155/2014/129208

**Published:** 2014-08-19

**Authors:** Marzieh Akbarzadeh, Zahra Masoudi, Mohammad Javad Hadianfard, Maryam Kasraeian, Najaf Zare

**Affiliations:** ^1^Community Based Psychiatric Care Research Center, Department of Midwifery, School of Nursing and Midwifery, Shiraz University of Medical Sciences, P.O. Box 71345-1359, Shiraz 71936 13119, Iran; ^2^School of Nursing and Midwifery, Shiraz University of Medical Sciences, P.O. Box 71345-1359, Shiraz 71936 13119, Iran; ^3^Department of Physical Medicine & Rehabilitation, School of Medicine, Shiraz University of Medical Sciences, P.O. Box 71345-1359, Shiraz 71936 13119, Iran; ^4^Maternal-Fetal Medicine Research Center, Department of Obstetrics & Gynecology, Shiraz University of Medical Sciences, P.O. Box 71345-1359, Shiraz 71936 13119, Iran; ^5^Department of Biostatistics, Infertility Research Center, School of Medicine, Shiraz University of Medical Sciences, P.O. Box 71345-1359, Shiraz 71936 13119, Iran

## Abstract

Delivery is considered as one of the most painful experiences of women's life. The present study aimed to compare the effects of supportive care and acupressure on the pregnant women's pain intensity and delivery outcome. In this experimental study, 150 pregnant women were randomly divided into supportive care, acupressure, and control groups. The intensity of pain was measured using Visual Analogue Scale (VAS). The supportive care group received both physical and emotional cares. In the acupressure group, on the other hand, BL32 acupoint was pressed during the contractions. Then, the data were analyzed using descriptive and inferential statistics. The results revealed significant difference among the three groups regarding the intensity of pain after the intervention (*P* < 0.001). Besides, the highest rate of natural vaginal delivery was observed in the supportive care group (94%) and the acupressure group (92%), while the highest rate of cesarean delivery was related to the control group (40%) and the difference was statistically significant (*P* < 0.001). The results showed that maternal supportive care and acupressure during labor reduced the intensity of pain and improved the delivery outcomes. Therefore, these methods can be introduced to the medical team as effective strategies for decreasing delivery pain. This trial is registered with the Iranian Registry of Clinical Trial Code IRCT2014011011706N5.

## 1. Introduction

Delivery is one of the most important phenomena of a woman's life. Delivery pain does not result from tissue trauma or damage; rather, it is a part of a unique physiological process [1]. The main factors in delivery pain are cervical dilatation and uterine contractions [2]. Severe delivery pain leads to the mother's emotional turmoil and disturbs her mental health. It also has several negative effects on maternal and fetal physiological status as well as the delivery progress, including increase of oxygen consumption, increase of pulmonary ventilation, increase of cardiac output, delayed gastric emptying, inefficiency of uterine contractions, prolonged labor, decrease in uterine perfusion, fetal hypoxia, and metabolic acidosis, leading to obstetric interventions and their resultant complications [3]. Consequently, fear and anxiety resulting from delivery pain increase the mother's pain and discomfort during the delivery process [4]. In the study performed by Lee et al. (2001), pregnant women mentioned severe pain as the most important factor in fear from delivery [5]. Thus, reduction of delivery pain is of great importance for decreasing the negative effects of the physiological processes which occur due to the mother's pain and anxiety and lead to maternal and fetal damages [6]. Up to now, various pharmacological and nonpharmacological methods have been used for sedating delivery pain. In the recent years, physicians and researchers have come to the conclusion that they should employ safe and effective methods which do not disturb the delivery process, mother's consciousness, and mother's straining reflex and other physiological actions in order to reduce pain. It should be noted that these complications are usually detected following utilization of pharmacological methods [7]. Moreover, nonpharmacological methods do not have any side effects for the mother and the fetus, do not interfere with the labor progress, and are even desirable for both the mother and her fetus [8]. One of these nonpharmacological methods is acupressure which is a kind of massage therapy developed in ancient China. Acupressure is a noninvasive method whose nature is similar to acupuncture [9]. This method is effective in sedating pain and reducing its intensity [10]. Some researchers believe that acupressure prevents transfer of pain stimulators, increases blood endorphin levels, and thereby reduces pain [11]. Chao (2007) and Chung (2003) have also emphasized the effectiveness of acupressure in reduction of labor pain [2, 11]. Overall, various acupoints are used for induction and reduction of labor pain. One of these points is BL32 which is located in the second hole of sacral bone [12].

Another nonpharmacological method for decreasing pain is accompaniment of a doula. Doula's supportive care includes emotional support (continuous presence, reassurance, and encouragement) and physical support (reduction of hunger, thirst, and pain, giving information about what is happening and how to deal with it, respecting the woman's decision, and helping her to create relationships with the caregivers) [13]. Supporting the mother has a considerable effect on reduction of labor pain. In this regard, Hofmeyr et al. indicated that supporting the mother by the doula caused the mothers to report less pain [14]. Also, Hodnett and Osborn stated that continuously supporting the mother by the doula during the labor significantly reduced utilization of analgesics [15]. Doula calms the mother down during labor and suggests different positions to increase fetal descent. Besides, doula's support leads to less utilization of oxytocin for augmentation, lower rate of instrumental delivery, less utilization of epidural anesthesia and narcotics, and lower rate of cesarean delivery [16].

Considering the harmful effects of severe delivery pain on mother, fetus, and delivery outcomes, safe and effective control of pain is of great importance. Nonetheless, a limited number of studies have been conducted on the effects of acupressure and supportive care on the delivery outcomes. Thus, the present study aims to compare the effects of maternal supportive care and acupressure at BL32 acupoint on the pregnant women's pain intensity and delivery outcomes.

## 2. Materials and Methods

This randomized clinical trial was conducted in the delivery ward of the selected educational center of Shiraz University of Medical Sciences (Shoushtari Hospital) in 2012. Considering *d* = 5, *α* = 0.05, 1 − *β* = 0.90, SD = 7, and the following formula, a 150-subject sample size (50 subjects in each group) was determined for the study:

(1) $$\begin{array}{c} n=\frac{2{\left(Z_{1-\alpha /2}+Z_{1-\beta }\right)}^{2}{\text{SD}}^{2}}{d^{2}}. \end{array}$$

Then, the subjects were selected through simple random sampling and were divided into supportive care, acupressure, and control groups using stratified block randomization.

The inclusion criteria of the study were 18–35 years of age, term pregnancy, singleton pregnancy, and healthy fetal membranes. Also, the study participants had no history of medical, surgical, or mental problems and had faced no special problems during pregnancy. The participants' uterine contractions started spontaneously and, at admission, the contractions occurred every 5–10 minutes and cervical dilatation was 3-4 cm. Written informed consent was obtained from all the study participants.

In the supportive care group, doula (the researcher) was constantly beside the mother from the beginning of the mother's maternity ward admission (beginning of the active phase of labor at 3-4 cm cervical dilatation) to the end of the second stage of labor. Supportive measures classified into psychological and emotional, educational, and physical categories were offered to the mother. Psychological and emotional support included touching, empathy, compassion, encouraging the mother to continue cooperation in the labor process, reassurance, taking mother's hands, maintaining eye contact, creating a sense of trust and confidence, continuous talking, and reduction of fear during labor. Educational support included informing the mother about the natural process of childbirth and answering her questions. Finally, physical support included cooling the mother, satisfying her hunger and thirst, and helping her change the positions in various stages of labor.

These positions were as follows: the mothers followed activity positions, such as straddling a chair, leaning, tailor stretching, and lunging for 20 minutes at 3–8 cm dilatation. Then, they were required to follow relaxing positions, such as semisitting and side-lying for 10 minutes. Since 8–10 cm dilatation, the mothers followed fetal head descent positions, such as dangling, squatting, and hands and knees.

**Figure 1:**
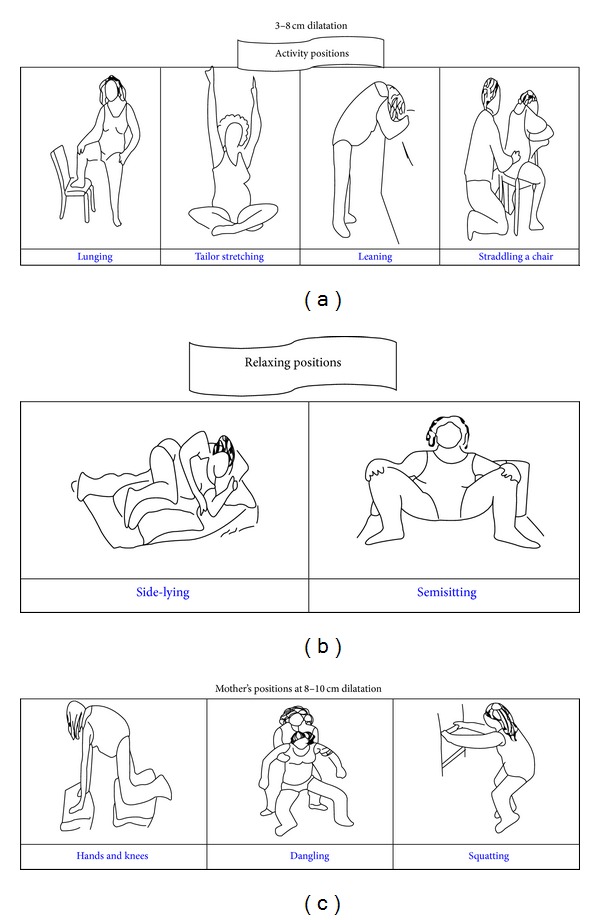
Maternal position during labor. Reference: http://www.childbirthconnection.org/pdfs/comfort-in-labor-simkin.pdf.

In this study, 20 minutes were considered for changing maternal positions during labor in order to avoid boredom and monotonous conditions for the mother (Figure 1).

**Figure 2:**
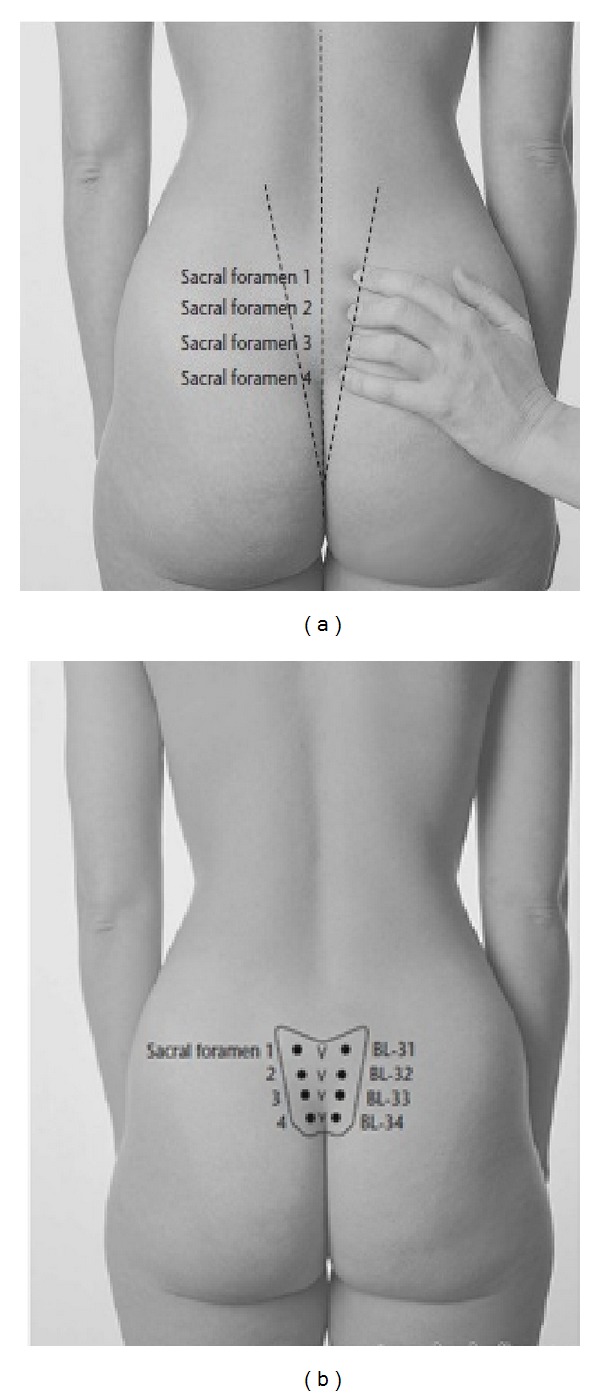
Location of the BL32 point. Reference: http://acupunctureschoolonline.com/bl-31%E2%80%93bl-34-eight-liao-baliao-acupuncture-points.html.

In the acupressure group, the participants were located in the appropriate position and BL32 acupoint was pressed. This acupoint is located in the second hole of sacral bone. This point lies approximately one index finger length above the top of the buttock crease, approximately one thumb width either side of the spine (Figure 2) [17]. Pressure was applied by the beginning of contractions (3-4 cm cervical dilatation) and continued during the transitional phase of labor (7-8 cm cervical dilatation) up to the end of the first stage. In doing so, when the contractions began, the aforementioned point was pressed gently for 30 seconds. The amount of pressure was determined by an electrical engineer who was familiar with such interventions using a digital device. The researcher attempted to apply a certain amount of pressure at each time. After the training, the pressure applied by the right and the left thumbs was measured as 1405 and 1277 mmHg, respectively. The pressure was applied by the beginning and stopped at the end of the contractions. To ensure that there was no difference in the amount of the pressure exerted by the right and left thumbs, the amount of pressure was computed using the related formula. The difference in applied pressure was minimized through repetition and practice. The intervention was performed for 30 minutes only during the contractions.

The control group only received the department's routine care and underwent no interventions.

The study data were collected using interview form (including demographic information, history of pregnancy, familial status, and pregnancy information), observation form (including evaluation of uterine contractions, fetal heart rate, labor progress, and delivery outcome), and Visual Analogue Scale (VAS).

VAS is a scale numerated from 0 to 10 with 0, 1–3, 4–6, 7–9, and 10 representing no, mild, moderate, severe, and the worst possible pain, respectively [18]. The validity and reliability of this scale have been confirmed in the study conducted by Molazem et al. in Iran. In that study, the content validity of VAS was determined using experts' opinions and its Cronbach's alpha coefficient was computed as 0.80 [19].

After all, the data were entered into the SPSS statistical software (v. 16) and analyzed using Wilcoxon nonparametric test, Chi-square test, and one-way ANOVA.

## 3. Results

In this study, the participants were similar regarding age (*P* = 0.496), number of pregnancies (1 or 2), cervical dilatation at admission (3-4 cm), gestational age (37–41 weeks) (*P* = 0.158), educational level (*P* = 0.584), and occupation (*P* = 0.781).

Considering the intensity of pain, the results of Wilcoxon nonparametric test showed no significant difference among the supportive care (6.14 ± 0.926), acupressure (6.52 ± 1.054), and control groups (6.20 ± 1.088) before the intervention (*P* = 0.354). After the intervention, however, the intensity of pain reduced in the supportive care (3.54 ± 1.328) and acupressure groups (3.44 ± 0.907) compared to the control group (9.40 ± 1.010) and the difference was statistically significant (*P* < 0.001). Nonetheless, no significant difference was observed between the two intervention groups concerning the intensity of pain (*P* > 0.05) (Table 1).

Moreover, the results of Chi-square test revealed a significant difference among the three groups regarding the mode of delivery (*P* < 0.001). Accordingly, the rate of natural vaginal delivery in the supportive care group was 2% and 34% higher than that in the acupressure and control groups, respectively. The highest rate of natural vaginal delivery was observed in the supportive care group (94%) and the acupressure group (92%), while the highest rate of cesarean delivery was related to the control group (40%). Overall, 18% of all the deliveries were carried out through cesarean section (Table 2).

## 4. Discussion

In this study, all the participants' pain intensity increased by increase in dilatation in the first stage of labor. Yet, the intensity of pain was lower in the two intervention groups compared to the control group and the difference was statistically significant (*P* < 0.001). According to Lowe (1996), the intensity of pain is extremely high in the first stage of labor, particularly in the transition phase (8–10 cm dilatation) [20]. Based on Western medicine, the intensity of uterine contractions is closely related to delivery pain. However, pharmacological interventions prevent effective uterine contractions. These medications increase uterine contractions, but they increase the delivery pain, as well. However, traditional Chinese medicine (TCM) has shown that acupressure can maintain balance during labor, reduce labor pain, and improve the delivery process by increasing the uterine contractions [21]. Chung et al. (2003) also reported that the participants of the acupressure group experienced less pain during labor compared to those of the touch and the control groups [11]. Reduction of pain by acupressure at BL32 acupoint can be justified by the gate control theory of pain. According to this theory, acupoints are the locations of sensory receptors with thin afferent fibers (A-delta and C-fibers) placed in the muscles. By stimulation of these points by needle, pressure, or transcutaneous electrical nerve stimulation (TENS), the sensory receptors are activated and send the stimulations to the spinal cord. In this way, the spinal cord, midbrain, and hypothalamic-pituitary axis are activated and present their analgesic effects through releasing enkephalin and endorphin [22].

In the present study, doula's continuous support significantly reduced the intensity of labor pain compared to the control group, which is consistent with the results of many studies conducted on the issue. For instance, the study by McGrath and Kennell (2008) showed that continuous support during labor considerably decreased the need for analgesics [23]. Also, the results of the research by Pascali-Bonaro demonstrated that supporting the mother during labor facilitated delivery and decreased the intensity of delivery pain [24]. Reduction of pain by this strategy can be justified by Melzack's neuromatrix theory. This theory provides a novel concept of a widely distributed neural network in reception and perception of pain on one hand and individuals' responding method and physical as well as psychological behavior on the other hand. According to this theory, pain is a multifactorial experience. Besides, based on the brain's view towards the whole body, it not only responds to the sensory input, but it can also experience pain without any stimulation or input from the sensory neurons [25]. Hence, presence of the doula, her psychological support, and suggestion of various positions during labor led to a decrease in mother's pain by changing her physical and psychological behavior.

The findings of the current study revealed a significant difference among the three groups concerning the mode of delivery (*P* < 0.001). The highest rate of natural and cesarean delivery was observed in the supportive care and the control groups, respectively. Doula can play a critical role in changing delivery into a desirable experience and reducing the rate of cesarean delivery by helping, supporting, consoling, and reassuring the women in labor and encouraging them to do physical activities [26]. In the study conducted by Zhang et al., the rate of vaginal delivery was 2-fold higher than that of cesarean delivery in the supportive group [27]. Similarly, Scott indicated that the rate of cesarean section decreased by 47% in the continuous support group in comparison to the control group [28]. In the studies by Hodnett [29], Klaus and Kennell [30], and Hatem et al. [31] also, the rate of cesarean delivery was higher in the control group compared to the supportive care group. All these findings are in agreement with those of the present study which showed the lowest rate of cesarean delivery in the supportive care group. All these researchers emphasized the effect of continuous support on reduction of labor pain. Of course, the current study had a major difference with the other ones. In this study, in addition to psychoemotionally supporting the women, doula suggested appropriate positions for opening the cervix at 3–8 cm dilatation and other positions for increasing fetal head descent at 8–10 cm dilatation. In the study by Bloom, 4 and 6 deliveries were carried out through cesarean surgery in the active and resting groups, respectively [32], but the difference was not statistically significant. Roberts et al. [33] and Lawrence et al. [34] also found no significant association between mother's position and type of delivery. The difference between the results of the present study and those of other studies might be due to the difference in mother's positions during labor. In this study, the effective positions in fetal head descent were recommended to the mother to reduce pressure on the inferior vena cava and improve oxygenation to the fetus. In bloom's study, on the other hand, the women only walked during labor. In contrast, Albers et al. indicated a significant relationship between mother's activity in labor and type of delivery [35]. In that study, the rate of cesarean delivery was 2-fold higher in the control group compared to the mothers who were active during labor (5.5% versus 2.7%). In the current study, the rate of natural vaginal delivery was significantly higher in the acupressure group in comparison to the control group and the highest rate of cesarean delivery was observed in the control group.

## 5. Conclusion

The study findings showed that continuous support and position change during labor as well as acupressure reduced the intensity of pain and the rate of cesarean delivery. Therefore, these two nonpharmacological methods can be used to improve the delivery outcomes and create a positive delivery experience.

## Figures and Tables

**Table 1:** Comparison of the mean intensity of pain in the three groups before and after the intervention.

	Group			
Pain assessment time	Supportive care (*n* = 50)	Acupressure (*n* = 50)	Control (*n* = 50)	*P* value
	M ± SD	M ± SD	M ± SD	
Before the intervention	6.14 ± 0.926	6.52 ± 1.054	6.20 ± 1.088	0.354
After the intervention	3.54 ± 1.328	3.44 ± 0.907	9.40 ± 1.010	0.000

Significance level: *P* < 0.05.

**Table 2:** Comparison of delivery mode in the intervention and control groups.

Group	Supportive care (*n* = 50)		Acupressure (*n* = 50)		Control (*n* = 50)		Total (*n* = 150)		*P* value
Delivery mode	Number	Percentage	Number	Percentage	Number	Percentage	Number	Percentage	
Vaginal delivery	47	94	46	92	30	60	123	82	0.000
Caesarean section	3	60	4	8	20	40	27	18	

Significance level: *P* < 0.05.
